# Editorial: Radiomics and artificial intelligence in oncology imaging

**DOI:** 10.3389/fnume.2026.1803820

**Published:** 2026-03-06

**Authors:** Vincent Habouzit, Jeppe Friborg, Morgan Michalet

**Affiliations:** 1 Department of Nuclear Medicine, University Hospital of Saint-Etienne, Saint-Etienne, France; 2 Department of Oncology, Copenhagen University Hospital - Rigshospitalet, Copenhagen, Denmark; 3 Department of Radiation Oncology, University Federation of Radiation Oncology of Mediterranean Occitanie, Montpellier Cancer Institute, Univ Montpellier, Montpellier, France; 4 INSERM U1194 IRCM, Montpellier, France

**Keywords:** artifical intelligence, nuclear medicine, oncology, radiology, radiomics

Over the past decade, radiomics and artificial intelligence (AI) have experienced rapid growth in the scientific literature and have raised considerable expectations for reshaping oncologic imaging. By integrating AI-driven analytics, these approaches move beyond subjective visual assessment to extract high-dimensional patterns from imaging datasets to improve clinical decision-making. This Research Topic brings together original studies and review articles that illustrate both the potential and the current limitations of radiomics- and AI-driven methodologies across a wide range of tumor types and imaging modalities.

## Improving cancer diagnosis and staging

One of the strongest messages emerging from this collection is the particular value of AI in diagnostically equivocal or “gray-zone” clinical situations. In such settings, several original contributions illustrate how AI-driven imaging models may support lesion characterization and disease stratification.

In lung imaging, Jha et al. reported robust performance of [^18^F]fluorodeoxyglucose positron emission tomography-computed tomography-based radiomics ([^18^F]FDG PET/CT) for the characterization of solitary pulmonary nodules, underscoring the added value of AI-augmented metabolic imaging in supporting early lung cancer diagnosis. Similarly, in thyroid imaging, studies by Guo et al. and Zhang et al., showed that ultrasound-based radiomics may improve the characterization of diagnostically challenging nodules, including sub-centimeter and partially cystic lesions. Benefits of radiomic models where also reported in prostate cancer screening with magnetic resonance imaging (MRI) within the “PSA gray zone” (4–10 ng/mL). In this context, Ji et al. showed that MRI-based radiomics could improve risk stratification compared with more conventional tools such as PI-RADS and PSA density.

Furthermore, diagnostic performances of AI-driven models could be reinforced by adding multimodality data together with imaging-derived features. Across tumor types, models integrating radiomics with clinical, biological or pathological variables consistently tended to outperform single-domain approaches. This is particularly notable in lung cancer, where Li et al. developped an ensemble learning model integrating [^18^F]FDG PET/CT radiomic features with clinical risk factors, which demonstrated strong performance for predicting lymph node metastases. Similarly, in papillary thyroid carcinoma, Feng et al. showed that combining clinical variables with ultrasound- and CT-derived features enabled accurate preoperative prediction of lateral lymph node metastases. Beyond incremental gains in predictive performance, these studies suggest that multimodal integration enables AI models to better reflect the multifactorial nature of cancer progression, thereby enhancing their robustness and clinical plausibility in real-world oncologic imaging applications.

## Virtual biopsy

Beyond diagnosis, this collection also addresses the broader use of AI-driven imaging as a non-invasive surrogates of tumor biology. For exemple, Qin et al. developped a CT-based deep learning radiomics model that successfully predicted Ki-67 expression levels in non-small cell lung cancer (NSCLC) while Xia et al. applied magnetic resonance imaging-based radiomics combined with machine-learning approaches to differentiate primary NSCLC from small cell lung cancer (SCLC) in patients with brain metastases. Similarly, Zhu et al. reported an ultrasound-based modeling approach for the non-invasive prediction of BRAF V600E mutation status in thyroid cancer. In a rarer cancer type, Wang et al. showed that MRI-based radiomics may enable preoperative prediction of soft tissue sarcoma grade, with potential implications for guiding neoadjuvant chemoradiotherapy decisions. By providing a non-invasive, whole-tumor assessment, such imaging-based approaches may complement the limitations of focal needle biopsy, particularly in the presence of intratumoral heterogeneity.

More broadly, this paradigm may support more dynamic disease assessment through repeated evaluation of intra-patient inter- and intra-lesional heterogeneity over time, and may help inform prognostic assessment beyond what is typically achievable with conventional tissue sampling.

## Predictive models of treatment response and toxicity

Treatment response prediction was another major focus of this research topic. In a systematic review and meta-analysis, Rodriguez et al. compared AI-based models with radiologist evaluation and reported a modest but statistically significant advantage of AI for predicting treatment response in lung cancer, particularly in CT and PET/CT imaging.

Radiomics was further applied to treatment-related toxicity prediction. In nasopharyngeal carcinoma, Hong et al. developed a combined machine-learning model integrating clinical, radiomic, and dosiomic features, which demonstrated higher predictive accuracy than single-domain models for acute radiation-induced dermatitis. Such approaches may enable pre-therapeutic risk stratification and support personalized preventive strategies.

## Perspectives and limits

Despite these encouraging results, several authors appropriately caution against overinterpretation. Although complex models integrating multidimensional data frequently demonstrated good performance across various clinical settings, their incremental clinical value compared with simpler, widely used clinical or radiological scores and decision-making algorithms remains insufficiently established. Moreover, many studies rely on retrospective, single-center cohorts, with limited external validation, thereby restricting generalizability. As an illustration, review articles focusing on gynecologic malignancies and osteosarcoma by Zhang and Qin and Zhang et al. highlight persistent challenges related to dataset heterogeneity, limited sample sizes, and the lack of standardized imaging acquisition and preprocessing pipelines.

While the growing use of interpretable approaches such as Gradient-weighted Class Activation Mapping (Grad-CAM), SHapley Additive exPlanations (SHAP), and Local Interpretable Model-agnostic Explanations (LIME) is encouraging, their adoption remains limited and inconsistently reported across studies, including those by Liu et al. and Feng et al.

Future research should prioritize multicenter prospective studies, standardized radiomics workflows, and formal comparisons with established clinical risk models. Economic evaluations and regulatory considerations will also be essential to support real-world implementation.

## Conclusion

This Research Topic illustrates a field transitioning from methodological exploration toward clinically meaningful integration. Radiomics and AI appear to show their greatest value in improving decision-making in complex and uncertain oncologic scenarios. With continued efforts toward standardization, validation, and interpretability, these approaches may play an increasingly important role in the future of precision oncology.

